# Free summer programming on elementary-aged children’s food and beverage consumption: a randomized clinical trial

**DOI:** 10.1186/s12966-025-01861-1

**Published:** 2025-12-20

**Authors:** Meghan C. Savidge, Sarah Burkart, Christopher D. Pfledderer, Elizabeth L. Adams, R. Glenn Weaver, Bridget Armstrong, Keith Brazendale, Xuanxuan Zhu, Brian Chen, Alexander McLain, Michael W. Beets

**Affiliations:** 1 Arnold School of Public Health, University of South Carolina, Columbia, SC USA; 2 UTHealth Houston, School of Public Health, Austin , TX USA; 3 Department of Health Sciences, University of Central Florida, Orlando, FL USA

**Keywords:** Poverty, Intervention, Diet, Children, Summer Vacation

## Abstract

**Purpose:**

During summer vacation, many children in households with low income lose access to federally-funded, healthful school meals. Summer day camps (SDCs) provide access to healthful meals and a structured environment; yet low-income families often cannot afford SDCs which may influence food/beverage consumption. This study examined the impact of receiving a free SDC versus experiencing summer as usual (SAU) on dietary intake during summer among children from low-income families.

**Methods:**

Parent-child dyads (*N* = 422; child age: 8.2 ± 1.5 yrs; 48% female; 51% Black) were recruited over 3 years (2021–2023) from schools serving families with low-income. Children were randomized to receive 8–10 weeks of free SDC (intervention) or SAU (control). Parents completed daily diaries for 14 days during school (April/May) and summer (July) which captured child consumption of healthful (e.g., fruit, vegetables, milk) and unhealthful (e.g., soda, fast food, snacks/chips) foods/beverages. Mixed-effects intent-to-treat (ITT) models examined the odds of consuming different foods/beverages during summer, controlling for school year consumption, in the SDC group compared to SAU. Secondary as-treated analyses examined the impact of attending structured summer programming versus not attending on the odds of consuming different food/beverages, regardless of randomization.

**Results:**

A total of 2,931 daily diaries were completed for intervention (*n* = 232) and control (*n* = 201) children. ITT analyses showed the SDC group had decreased odds of consuming frozen desserts (OR 0.68, 95%CI 0.49–0.94), compared to SAU, during summer weekdays. No other differences were observed. As-treated analyses showed children had increased odds of consuming fruit (1.77, 1.30–2.41), milk (1.97, 1.44–2.69), and chips/snacks (1.49, 1.17–1.90), and decreased odds of consuming soda (0.53, 0.34–0.84), fast food (0.57, 0.45–0.73), and frozen desserts (0.58, 0.45–0.74) on weekdays when they attended structured summer programming, compared to days they did not attend.

**Conclusions:**

Providing free SDC alone did not promote more healthful food/beverage consumption during summer vacation compared to SAU. However, on days children attended a structured program, children experienced more dietary benefits compared to days they did not. Thus, structured environments may positively impact children’s diet during summer; yet, identifying strategies to address barriers beyond cost to improve attendance and enrollment may enhance the impact of summer programming.

**Supplementary Information:**

The online version contains supplementary material available at 10.1186/s12966-025-01861-1.

## Introduction

Children gain 3–5 times more weight during the 3 months of summer vacation compared to the entire school year [1–3]. Notably, the prevalence of accelerated summer weight gain is highest in children from families with low income [4, 5]. While many factors influence summer weight gain and thus overweight/obesity (OW/OB) risk, dietary changes from school to summer vacation are of potential concern. During the school year, >30 million children from households with low income are provided federally funded nutrient-rich meals at consistent and structured times with regulated portion sizes each day [6, 7]. Evidence shows school meals, provided by the National School Lunch Program (NSLP), have improved over time and provide a higher nutritional quality (e.g., lower sodium, more fruit/vegetables) than meals obtained elsewhere, such as at home where dessert foods, sugar sweetened beverages, and snack chips may be more readily available [8–12]. At the start of summer break, millions of children lose access to nutrient-rich, school provided meals, resulting in a larger proportion of dietary intake that must come from home and/or other sources providing low-cost, energy dense options (e.g., fast food, convenience stores, community programs) [6, 13]. Of the few studies that have examined dietary changes from the school year to the summer vacation, the findings are mixed; yet, the majority of these studies suggest a decrease in consumption of fruit and vegetables, as well as an increase in added sugar during the summer [5, 14–19]. Thus, there is a need to evaluate interventions that could maintain more healthful dietary intake during the summer [14, 17, 19]. 

According to the Structured Days Hypothesis, a structured summer environment (i.e., a child attends a program or camp during summer) that provides federally regulated meals and limits opportunities for added meals/snacks, similar to the school year, is expected to promote more healthful dietary intake compared to less structured summer environments, such as home [20–22]. During the summer, the absence of the school day reduces children’s exposure to a nutritionally regulated food environment, leading to increased consumption of foods and beverages in unstructured settings [1, 20]. One potential solution to maintain access to structured environments that provide nutrient-rich, federally funded meals during the summer is to leverage existing summer programs, such as summer day camps (SDC) [23–25]. Traditional SDCs operate from 8:00am-5:00pm, Monday through Friday, for approximately 10 weeks. SDCs may provide up to three balanced meals each day adhering to United States Department of Agriculture (USDA) guidelines; however, meal offerings may vary across SDCs and other structured summer programs [26, 27]. Camps operating in areas where at least half of the children are from households with low income (≤ 185% federal poverty level), are eligible to operate as Summer Food Service Program (SFSP) sites, which allows reimbursement for meals provided to all children [22]. Regardless of SFSP participation, the provided structure SDCs provide around meals limits additional opportunities to eat/drink during the day, mirroring the school food environment [28]. However, most SDCs are an out-of-pocket expense for families, and children from households with low income are the least likely to attend SDCs, given the high cost [29–31]. This may contribute to disparities in accessing healthful meals and meal structure during the summer months.

There is limited understanding around how attending structured summer programming may impact specific food/beverage intake for children from households with low income. Previous studies report a more healthful diet quality, increased fruit consumption, and a protective effect on total energy intake when children attend structured summer programs compared to not attending [18, 32–34]; yet, these studies are limited by small sample sizes and lack a rigorous study design (e.g., single-arm, quasi-experimental). This study aimed to overcome these limitations by leveraging data from a large-scale randomized controlled trial (RCT) that examined the impact of providing access to free SDC (intervention), compared to summer as usual (control), in a sample of elementary-aged children from families with low income. The primary outcome of the RCT was changes in child Body Mass Index (BMI) z-scores [35]. This study presents an evaluation for the secondary outcome of parent-reported food/beverage consumption between children receiving free SDC (intervention) and summer as usual (control) during summer vacation as part of the larger RCT [35]. It is hypothesized that children randomized to receive free SDC will consume more healthful foods/beverages and less unhealthful foods/beverages in summer compared to children randomized to summer as usual. Further, this study examined the impact of attending structured summer programming, including SDC and alternate structured summer programming, versus not attending on the odds of consuming different food/beverages, regardless of random group assignment. It is hypothesized that on days children attend structured summer programs, they will consume more healthful foods/beverages and less unhealthful foods/beverages in summer compared to days they do not attend.

## Methods

The RCT collected dietary data on elementary-aged children during the spring and summer of 2021, 2022, and 2023. The RCT was registered at Clincaltrials.gov (NCT04072549) and followed the Consolidated Standards of Reporting Trials (CONSORT) reporting guidelines [36]. Institutional Review Board approval was obtained prior to the start of data collection.

### Study design and participants

Children were recruited from 7 Title 1 elementary schools within a single school district in the southeastern United States. These schools serve ≥ 80% of children from families with low income. In the spring of each school year (March-April), study recruitment materials (i.e., paper flyers, online newsletter, Class Dojo messages) were distributed to caregivers of children enrolled in kindergarten through 4th grade at the partnering elementary schools. To participate, children were required to be enrolled in one of the partner elementary schools, had no plans to move during the summer, and were available to complete study assessments in both the spring and summer (i.e., not absent during assessment periods). Informed consent was obtained from one parent/guardian through an online Health Insurance Portability and Accountability Act (HIPAA)-compliant survey.

### Randomization and blinding

Upon completion of the informed consent and an income proxy screener in which parents reported the use of assistive services (i.e., WIC, SNAP, Medicaid), children were randomized 1:1 to either receive free SDC (intervention) or summer as usual (control). Randomization was stratified by number of public assistance services received (e.g., SNAP, WIC), child grade (kindergarten to 4th), and child biological sex. In cases where siblings were enrolled, children from the same household were grouped together and randomized to the same condition. Randomization occurred prior to baseline assessments to ensure ample time to arrange for summer childcare for those receiving summer as usual. A co-investigator who was external to the day-to-day conduct of the trial randomized children. The lead investigator and data collectors were blinded to group assignment.

### Intervention and control conditions

Children randomly assigned to receive free SDC (intervention) were offered 8–10 weeks of camp. Due to COVID-19 school closures, the summer of 2021 was shorter than the summers 2022 and 2023, resulting in only 8 weeks of free SDC that year. The RCT partnered with an existing SDC operated by a local parks and recreation commission with no outside influence from the study team. This organization has a history of operating camps in the community and upholds the necessary safety and staff training certifications to provide an optimal environment [25, 37]. Transportation to and from camp was provided during summer 2022 and 2023 for intervention children; however, transportation was not provided in 2021 due to COVID-19 restrictions. The SDC operated 7:00am-5:00pm Monday-Friday for 8–10 weeks of summer, except for the 4th of July holiday week. The SDC provided designated time for outdoor and indoor physical activity (3–4 h/day), enrichment, academic programming, and weekly field trips (4–5 h/day) to various community parks and entertainment settings (e.g., movies, bowling). Breakfast, lunch, and snacks, adhering to the SFSP, were provided to children at set times each day and reimbursed to camps through existing federal food programs [38]. For a meal to be reimbursed, the SFSP required breakfast to include one serving of milk, fruit/vegetable, and grains/bread [22]. Lunch had to include one serving of milk, two servings of fruits/vegetables, one serving of grains/bread, and one serving of a meat/meat alternative [22]. Snacks had to include at least one serving of two of the following food components: milk, fruits/vegetable, grains/bread, and meat/meat alternative [22]. SDCs had to meet the minimum required serving sizes for reimbursement, with additional servings usually offered to older children and reimbursed at the same rate [22]. The SDC participating in this study did not restrict children from bringing outside food to camp for consumption. An example SDC menu for breakfast, lunch, and snack is provided in Supplementary Materials Fig. 1. Children randomized to the control group did not receive free SDC, but experienced “summer as usual” as standard care in the summer (see Structured Program Exposure below).

## Measures

### Food and beverage consumption

Children’s food/beverage consumption was obtained via parent-reported daily diaries for 14 days during the spring (April/May) and 14 days during summer (July). Within the daily diaries, parents completed a modified version of the 2009–2010 National Health and Nutritional Examination Survey (NHANES) Dietary Screener Questionnaire (DSQ) [39]. The DSQ is a 26-item food frequency questionnaire in which parents report the frequency of foods/beverages consumed by their child each day, including fruits and vegetables, dairy/calcium, sugar-sweetened beverages, sweets/desserts, whole grains, red meat, and processed meat. For this study, questions on meat were excluded as there is no strong association between meat consumption and childhood obesity, while a question around snacks/chips was added to best reflect the diet of children [40]. Parents were sent the daily diary at 8:00pm each day and were asked to complete this with their child to report on what their child ate and/or drank that day. Unlike the original screener, which asked respondents about consumption over the past 30 days (e.g., 1 time last month, 2 times per week), only response options referring to the frequency of consumption for that day were included (e.g., 1 time today, 2–3 times today) to capture day to day variation in consumption of each food/beverage component. For foods items, response options were “none”, “1 time per day”, and “2 or more times per day”. For beverages, the response options were “none”, “1 time per day”, “2–3 times per day”, “4–5 times per day”, and “6 or more times per day”. Procedures for assessing daily food/beverage consumption used in this study were similar to previous investigations with similar populations [40]. The average response across the beverage items was 0.77 ± 0.92/day (range 0 to 3). For food items, the average response was 0.92 ± 0.78/day (range 0 to 2). During analysis, response options were collapsed and recoded as did eat/consumed or did not eat/did not consume due to a non-normal distribution in responses and low endorsement of higher consumption categories.

### Child and parent demographics

During each school year (April/May), parents completed an electronic survey, texted to their smart phone, to collect child biological sex, child race/ethnicity, household income (reported in $10,000 increments), parent’s highest education level obtained, household food insecurity status (food insecure, food secure) [41], and the use/receipt of any of the following public assistance services: Welfare, Temporary Assistance for Needy Families (TANF), Temporary Cash Assistance (TCA), Children’s Health Insurance Program (CHIP), Medicaid, Supplemental Nutrition Assistance Program (SNAP), Women, Infants and Children (WIC), or Supplemental Security Income (SSI).

### Structured program exposure – time use record

Children randomized to receive free SDC (intervention) may not have attended or could have attended alternative programming options. Children randomized to receive summer as usual (control) may have paid out of pocket to attend structured summer programming, such as SDC. It was anticipated that approximately 20% of the children randomized to summer as usual would attend some type of summer programming based on national data [42]. To account for differential exposure to structured programing during the summer, parents reported the amount of time (e.g., minutes, hours, days) that their child spent at a structured summer program within the daily diary each night. Questions about program exposure were developed by the study team and asked about their child’s behaviors over the previous 24 h. For example, parents were asked what time their child went to bed the previous night and woke up that morning, whether their child left the home and where they went, and if any/which meals were consumed outside of the home. This data was used to estimate structured summer program exposure (minutes/day) and attendance (attended vs. not attended that day).

### Statistical power

Sample size calculations for this RCT were originally based on the ability to detect a statistically significant group (intervention vs. control) by time (before summer to end of summer) interaction in the primary outcome of age and sex specific BMI z-score [43] using a mixed-effects model with statistical power of 0.80 and an alpha of 0.05. Accounting for the intraclass correlation at the school level (0.06) and an anticipated attrition rate of 25%, the total number of children required to be enrolled at baseline was 420 resulting in a final sample of 330 (165 per condition) [44]. 

### Statistical analyses

The primary analysis was intent-to-treat (ITT), defined as using all available data from children according to the original group they were randomized to (free SDC or summer as usual) regardless of their parent-reported attendance at a summer program [45]. Mixed effects models were used to account for the nesting of repeated daily measures within children. The analyses examined the odds of consuming different foods/beverages during weekdays in the summer, controlling for school year endorsement of consuming that food or beverage, in the free SDC group compared to summer as usual. During analysis, day-level food/beverage response options (e.g., 1 time today, 2–3 times today) were collapsed and recoded as binary values: 1 = consumed and 0 = did not consume. Analyses excluded weekend days as the intervention was only delivered on weekdays. Missing data for outcome variables were accounted for using full information maximum likelihood estimation, allowing for all children’s data to be used regardless of missing data [46]. Covariates included child biological sex, parent education, food security status, the use of assistance services (e.g., SNAP, WIC), and income. Missing data for covariates was handled using multiple imputation of 100 datasets for both continuous and categorical covariates.

Secondary analyses were as-treated, defined as using all available data from children based on their parent-reported attendance at a structured program on weekdays in the summer, regardless of what group they were randomly assigned to [47]. This analysis used the same mixed models approach to account for the nesting of repeated daily measures within children. This analysis examined the impact of attending structured summer programming, both as a binary predictor (days children attended vs. days not attended) and as categorical minutes attended (30–239 min/d or ≥ 240 min/d vs. did not attend “zero” minutes [reference group]) on the odds of consuming different food/beverages during weekdays in the summer, controlling for consumption during the school year and all covariates included in the primary analysis. The 30–239 min/d grouping corresponds to half-day or shorter programs, while ≥ 240 min/d corresponds to longer than half-day up to full-day programs. The assumptions for all models were evaluated by examining multicollinearity, residual plots, and the distribution of random effects; no violations were found. STATAv17.0 was used to run all statistical analyses.

## Results

### Participants

**Fig. 1:**
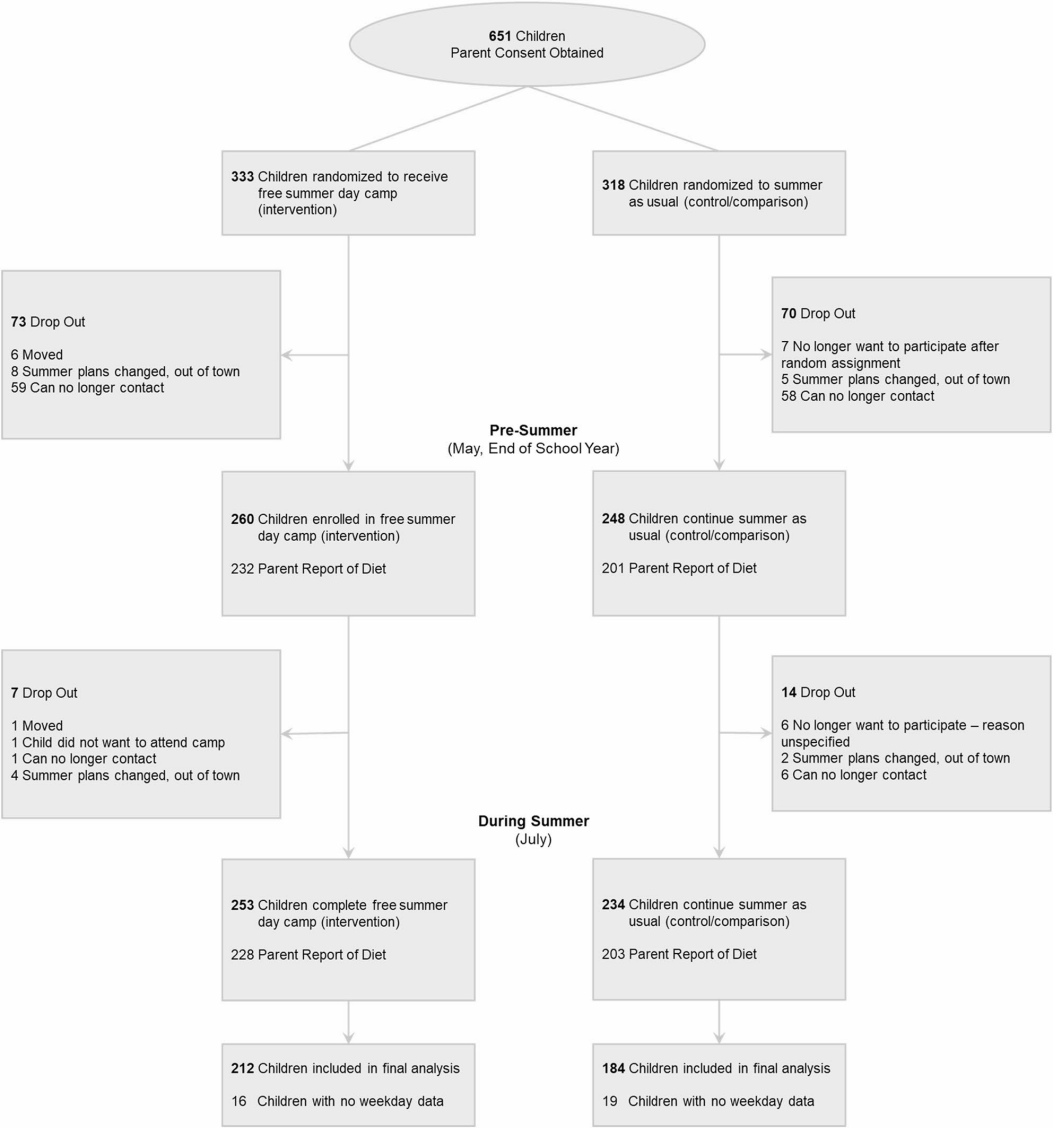
CONSORT Diagram

Of the 651 parents who expressed interest in participating in this study, 525 were contacted and randomized, with 260 randomized to receive free SDC and 248 randomized to summer as usual (Fig. 1). A total of 2,931 daily diaries (weekdays only) were completed for intervention (*n* = 211) and control participants (*n* = 183) during the summer timepoint, corresponding to summer dietary data collected on 81% of SDC (intervention) participants and 74% of summer as usual (control) participants. The demographic information of enrolled children can be found in Table 1.

**Table 1:** Demographics of children with dietary data during summer weekdays

	Control (SummerAsUsual)		Intervention (Free Summer Day Camp)	
Sample Size (n)	183		211	
Child Characteristics				
Female (n, %)	91,	49	99,	47
Age, mean ± SD, years	8.37	± 1.53	8.31	± 1.56
Ethnicity (n, %)				
Hispanic	14,	8	24,	11
Race (n, %)				
Black	86,	47	107,	51
White	71,	39	84,	40
Other Race^a^	6,	3	4,	2
Multiple Races	20,	11	16,	7
Household or Family Characteristics (n, %)				
Food Insecure	62,	35	57,	30
At or Below 200% Federal Poverty Level	110,	61	139,	68
Yearly Family Income (n, %)				
<$30k	45,	25	65,	31
$30k to $60k	67,	37	76,	37
>$60k	67,	37	67,	32
Services Received (n, %)^b^				
Welfare, Temporary Assistance for Needy Families (TANF), or Temporary Cash Assistance (TCA)	1,	1	2,	1
Children’s Health Insurance Program (CHIP), Medicaid	78,	43	95,	46
Supplemental Nutrition Assistance Program (SNAP)	48,	27	55,	29
Women, Infants and Children (WIC)	16,	9	22,	13
None	54,	30	50,	24
Parent Education (n, %)				
Some college or less	82,	45	111,	53
2 to 4 year degree	63,	35	69,	33
Graduate degree	37,	20	30,	14

^a^ Includes Asian, American Indian or Alaska Native, Native Hawaiian or Pacific Islander

^b^ Question was select all that apply, therefore the total percentage may be > 100%

### Intent-to-Treat (ITT) analyses

**Fig. 2:**
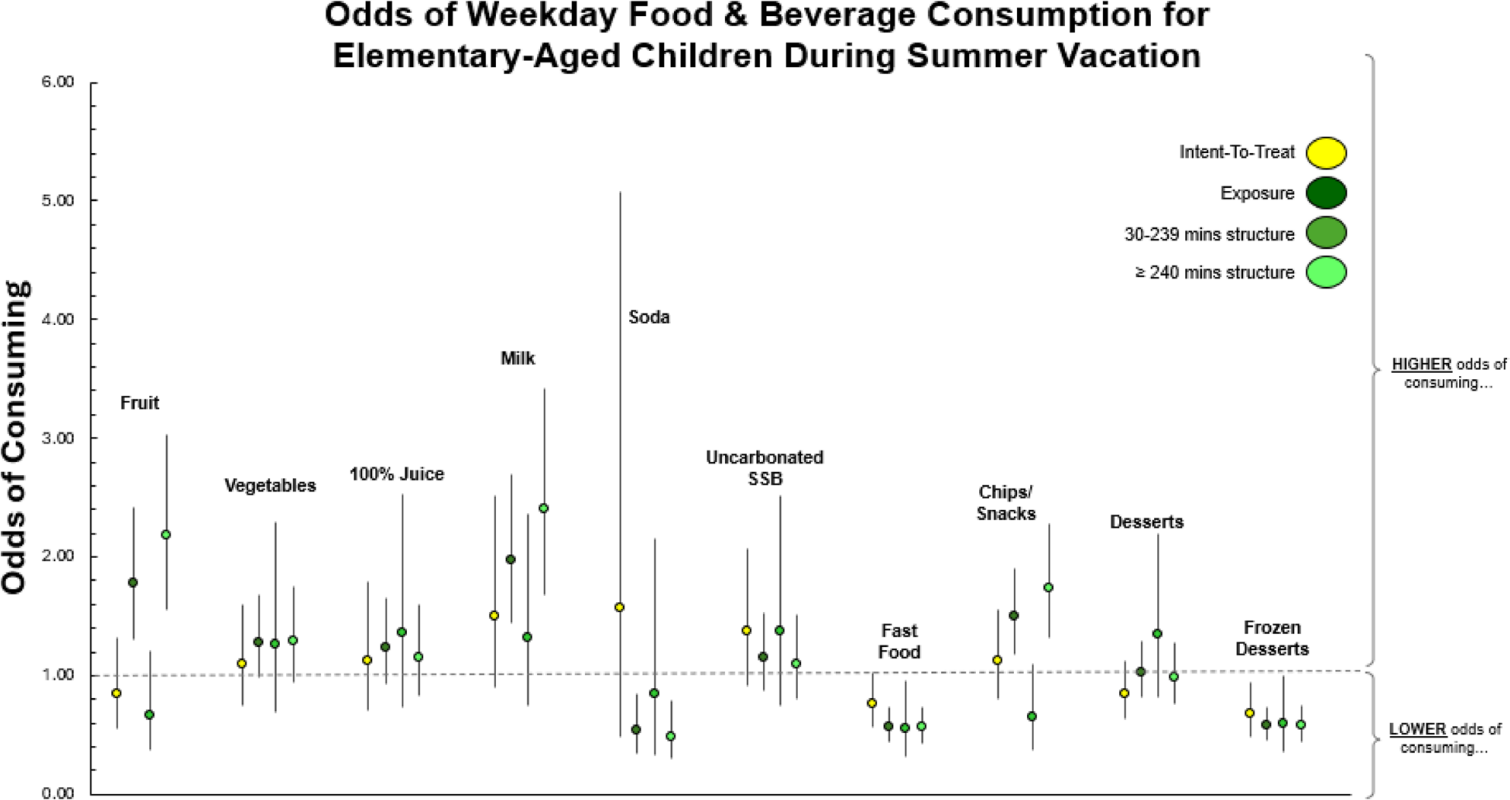
Odds of weekday food and beverage consumption for elementary-aged children during summer vacation Intent-to-Treat Reference Group: summer as usual (SAU) Exposure Reference Group: no structured programming (Intent-to-Treat) Differences in food/beverage consumption between children randomized to receive free SDC compared to children receiving SAU (Exposure) On days children attended structured programming, they had higher/lower odds of food/beverage consumption compared to days they did not attend (Exposure: 30–239 min) On days children attended 30–239 min of structured summer programming, they had higher/lower odds of food/beverage consumption compared to days they did not attend (Exposure: *≥*240 min) On days children attended *≥* 240 min of structured summer programming, they had higher/lower odds of food/beverage consumption compared to days they did not attend

ITT results for all foods/beverages can be found in Table 2. Children randomized to receive free SDC had lower odds of consuming frozen desserts (OR 0.68, 95%CI 0.49–0.94) compared to children randomized to receive summer as usual during weekdays in the summer when controlling for school year consumption (Fig. 2). No other significant differences were observed.

**Table 2:** Between group differences in odds of consuming foods/beverages during weekdays in the summer among elementary-aged children

Comparison	Primary Analysis			Exposure (Secondary Analysis)								
	Intent-To-Treat			Attend or NOT Attend Structured Programming (dichotomous)			30–239 min (Time in Structure)			≥ 240 min (Time in Structure)		
	Odds Ratio	(95CI)		Odds Ratio	(95CI)		Odds Ratio	(95CI)		Odds Ratio	(95CI)	
Foods & Beverages												
Healthful												
Fruit *(e.g.*,* Fresh*,* Frozen*,* Canned)*	0.85	(0.55,	1.32)	**1.77**	**(1.30**,	**2.41)**	0.67	(0.37,	1.21)	**2.17**	**(1.56**,	**3.03)**
Milk *(e.g.*,* Regular*,* Flavored*,* Lactose Free)*	1.50	(0.90,	2.52)	**1.97**	**(1.44**,	**2.69)**	1.32	(0.74,	2.36)	**2.40**	**(1.68**,	**3.43)**
100% Juice *(e.g.*,* Orange*,* Mango*,* Pineapple)*	1.12	(0.70,	1.79)	1.24	(0.92,	1.65)	1.36	(0.74,	2.52)	1.15	(0.83,	1.59)
Vegetables *(e.g.*,* Leafy Greens*,* Potatoes*,* Beans)*	1.10	(0.75,	1.60)	1.28	(0.98,	1.68)	1.27	(0.70,	2.30)	1.29	(0.95,	1.74)
Unhealthful												
Soda *(containing sugar)*	1.57	(0.48,	5.08)	**0.53**	**(0.34**,	**0.84)**	0.84	(0.33,	2.15)	**0.49**	**(0.30**,	**0.80)**
Uncarbonated Flavored Drinks *(e.g.*,* Kool-Aid*,* Lemonade*,* Gatorade)*	1.38	(0.92,	2.07)	1.16	(0.88,	1.53)	1.37	(0.74,	2.52)	1.10	(0.80,	1.51)
Fast Food *(e.g.*,* McDonalds*,* Bojangles*,* Sonic)*	0.76	(0.57,	1.03)	**0.57**	**(0.45**,	**0.73)**	**0.55**	**(0.32**,	**0.95)**	**0.56**	**(0.43**,	**0.73)**
Chips/Snacks *(e.g.*,* Chips*,* Pretzels*,* Crackers)*	1.12	(0.81,	1.55)	**1.49**	**(1.17**,	**1.90)**	0.64	(0.38,	1.10)	**1.73**	**(1.32**,	**2.28)**
Desserts *(e.g.*,* Chocolate*,* Doughnuts*,* Cookies)*	0.85	(0.63,	1.13)	1.03	(0.82,	1.29)	1.34	(0.82,	2.19)	0.99	(0.77,	1.28)
Frozen Desserts *(e.g.*,* Ice Cream)*	**0.68**	**(0.49**,	**0.94)**	**0.58**	**(0.45**,	**0.74)**	0.60	(0.36,	1.00)	**0.57**	**(0.44**	**0.74)**

Bolded values represent statistically significant results

All models adjusted for child biological sex, parent education, food security status, the use of assisted services (e.g., SNAP, WIC), income, and school year consumption

Intent-To-Treat reference group: summer as usual (child level)

Exposure reference group: days with 0 min of structured programming (day level)

### Exposure to structured program (as-treated analyses)

Children in both the free SDC and summer as usual group reported attending structured programming on weekdays in the summer (Table 3). Children randomized to free SDC attended more weekdays with at least 240 min of summer programming (60% of summer days, 900 out of 1502 days) compared to children randomized to summer as usual (17% of summer days, 246 out of 1103 days). Children randomized to free SDC attended fewer weekdays of shorter-duration (30–239 min) summer programming (3% of summer days, 52 out of 1502 days), compared to children randomized to summer as usual (6% of summer days, 80 out of 1429 days). Lastly, children randomized to free SDC did not attend a summer program on 37% of summer weekdays (550 out of 1502) compared to children randomized to summer as usual who did not attend a program on 77% of summer weekdays (1103 out of 1429).

**Table 3:** Weekday summer program attendance among children receiving free summer day camp or summer as usual

	Free Summer Day Camp		Summer As Usual	
	#	%	#	%
Total Weekdays Attended	952	63	326	23
*<240 min/day* ^*a*^	52	3	80	6
*≥240 min/day* ^*a*^	900	60	246	17
Weekdays NOT Attended	550	37	1103	77
Total Weekdays	1502	100	1429	100

^a^ # within Total Weekdays Attended, % out of Total Weekdays

Total Weekdays # and % = Total Weekdays Attended + Weekdays NOT Attended

The exposure (as-treated) analyses for foods and beverages are presented in Table 2. For the dichotomous variable of attended structured summer programming versus not attended, regardless of group assignment, children had statistically higher odds of consuming fruit (1.77, 1.30–2.41), milk (1.97, 1.44–2.69), and chips/snacks (1.49, 1.17–1.90), and had lower odds of consuming soda (0.53, 0.34–0.84), fast food (0.57, 0.45–0.73), and frozen desserts (0.58, 0.45–0.74) on weekdays when they attended any length of structured programming, compared to weekdays they did not attend structured programming (Fig. 2). On weekdays when children spent 30–239 min (half day or less) in structured programming they had statistically lower odds of consuming fast food (0.55, 0.32–0.95) compared to weekdays they did not attend structured programming (Fig. 2). On weekdays when children spent ≥ 240 min (> half day) in structured programming they had statistically higher odds of consuming fruit (2.17, 1.56–3.03), milk (2.40, 1.68–3.43), and chips/snacks (1.73, 1.32–2.28), and statistically lower odds of consuming soda (0.49, 0.30–0.80), fast food (0.56, 0.43–0.73), and frozen desserts (0.57, 0.44–0.74) compared to weekdays they did not attend structured programming (Fig. 2).

## Discussion

This study examined the impact of receiving free SDC on food/beverage consumption of elementary-aged children from families with low income during summer vacation. Guided by the Structured Days Hypothesis, it was expected that the consistent availability of federally funded healthful meals and limited opportunities for added meals/snacks would support more healthful eating patterns for children receiving free SDC compared to summer as usual. The ITT results indicated that offering free SDC alone does not promote more healthful food/beverage consumption during summer vacation. In fact, children receiving free SDC did not have increased odds of consuming more healthful food/beverages but had decreased odds of consuming frozen desserts (e.g., ice cream, popsicles) when compared to children receiving summer as usual. Yet, when children attended structured programming for more than a half day, greater dietary benefits were observed.

The limited impact of this intervention (free SDC) on children’s food/beverage consumption may be primarily attributed to a lower-than-expected attendance to free SDC, due to child sickness, planned family vacations, and/or changes in summer living/caregiving arrangements (e.g., staying with other parents or family members). Additionally, there was a higher-than-expected engagement in structured programming from the control group. Previous studies investigating the impact of structured programs on child diet report mixed findings with some studies reporting a positive impact on energy intake while others report no significant impact on food/beverage consumption or energy intake, though attendance rates were similar [18, 33, 48, 49]. In this study, children randomized to receive free SDC attended 60% of camp days offered (906 out of 1502), reducing their overall exposure to structured environments that consistently offer healthful meals, compared to if they had higher attendance at SDC. However, when they attended, most children attended for ≥ 240 min/day, allowing for potential dietary benefits. Greater attendance may have led to a stronger intervention effect. Further, when children randomized to the summer as usual condition attended a structured summer program of their own accord, they attended 17.7% of days, (253 out of 1103), also typically for ≥ 240 min/day, which was higher than anticipated but overall less than the intervention group. This unanticipated exposure to structured environments may have increased access to regulated meals and contributed to a crossover effect.

The exposure (i.e., as-treated) analysis showed that on days when children in either group attended structured summer programming (i.e., any programming vs. no programming), they had higher odds of consuming milk and fruit that day, consistent with SFSP guidelines which require milk to be offered for both breakfast and lunch at sponsored programming [22]. Additionally, children had increased odds of consuming chips/snacks (e.g., chips, pretzels, popcorn, etc.). While this is not a healthful impact of attending SDC, it is not unexpected, as chips and similar snack items are a kid-friendly, shelf-stable, inexpensive, and reimbursable item for SDCs participating in the SFSP [22]. Consequently, these items are commonly offered and consumed within SDCs, including the SDC utilized in this study [50]. An example menu for this SDC is provided in Supplementary Materials Fig. 1. Further, children were not restricted from bringing a meal or snack to camp and were not required to consume SDC food. This could have influenced the results; yet, information on whether children ate camp-provided food or home-provided food was not collected. When examining exposure based on the amount of time (0 min, 30–239 min, or ≥ 240 min) spent in a structured program, children had significantly higher odds of consuming healthful foods/beverages (e.g., fruit and milk) and significantly lower odds of consuming unhealthful foods/beverages (e.g., soda, fast food, and frozen desserts) when attending for ≥ 240 min. These findings indicate that a full day of programming resulted in greater dietary benefits than shorter durations (e.g., half day or less) and no programming. The reduced odds of consuming soda, fast food, and frozen desserts are favorable as these foods/beverages are dietary risk factors for OW/OB, increased BMI, and waist circumference [51, 52]. Fast foods and desserts tend to be high in added sugars, saturated fats, and calories, thus a high consumption of these foods could lead to increased risk of OW/OB [53]. Additionally, these foods are most commonly served at home [53]; thus children likely had less access and limited opportunities to consume these energy-dense nutrient-poor foods the longer they spent at day camp. The increased odds of fruit and milk consumption align with SFSP guidelines [22] and are similar to previous research [32, 34]. Increased fruit and milk consumption is promising as studies suggest diets high in fruit and/or milk is associated with a decreased risk of OW/OB in elementary aged children [51, 54, 55]. A regular consumption of fruit also supports vitamin, minerals, and fiber intake [38]. Furthermore, most children do not meet the recommended intake of fruit or dairy and tend to deviate further from the Dietary Guidelines for Americans as they age [38]. Thus, increased exposure to and consumption of these healthful foods and decreased consumption of unhealthful foods in early childhood is important for promoting lifelong healthful eating habits.

These results highlight the importance of maintaining a structured environment during the summer break. Additionally, when attending a structured program, attending for longer durations may lead to a more healthful dietary intake when compared to not attending a structured program. The more time children spend in a structured summer program, the more structured meals children receive, resulting in greater overall impact on food/beverage consumption. For example, full-day summer camps serve up to 3 meals (e.g., breakfast, lunch, dinner) or 2 meals and 1 snack (e.g., breakfast, lunch, snack) on site. Yet, if children attend for less than a full day, they do not receive the meals or snacks they missed when not in attendance [56]. In this study, the exposure analysis showed that on days when children attended a full day program, meaning they received up to 3 meals/snacks consumed in a structured environment that day, had the greatest dietary benefit. These findings illustrate not only the positive impact that SDC can have on child dietary intake, but the specific advantage to full day programs, where a greater proportion of children’s meals are served in a structured environment.

Overall, the exposure analyses provide evidence that structured summer programs play a meaningful role in shaping children’s food environments during the summer. Specifically, when children attend full-day programs that limit unscheduled eating occasions and offer healthful meals and snacks, their dietary intake is more healthful. Broader support for structured summer opportunities, such as reducing or removing the cost barrier (i.e., discounts, scholarships, vouchers), is needed to increase access to structured programs, as some families may not have had the opportunity to attend otherwise. However, providing access through the removal of costs alone is not enough, as evidenced by the ITT analyses. Consistent attendance and enrollment are critical to meaningful dietary benefits. To maximize the benefits of structured summer programming, it is essential to identify additional barriers to attending and enrolling in these programs beyond cost (e.g., child interest, scheduling conflicts, transportation) to implement comprehensive interventions and policies around structured summer programming and diet [31]. Moreover, challenges extend beyond families to the programs themselves. The number of programs participating in the SFSP continues to decrease with ~ 12,000 sites fewer participating compared to pre-COVID-19, largely due to staffing and systemic barriers [57]. Increased support for summer programs to (re)enroll in the SFSP is needed to maintain access to healthful summer meals for enrolled children. Future research should examine the potential dietary impact of other SFSP providers (e.g., community centers, schools, libraries) as well as other geographic locations (e.g., states, cities, communities) which may differ in service availability. Additionally, understanding parent and child perspectives of summer program meals and barriers to accessing meals or healthful foods during the summer would inform more effective program and policy strategies aimed at improving summer dietary intake.

### Limitations and strengths

This study was carried out in one mid-sized city in the southeastern United States, which may result in findings not generalizable to other geographical regions with varying characteristics. Additionally, only one existing SDC was leveraged which serves > 50% children from households with low income, and the operation of this SDC may not be generalizable to all SDCs or structured summer programs outside of this region and which serve differing populations. However, the partnering organization has long-standing experience operating numerous SDCs in the area. As such, the camp included in this study is likely representative of similar programs implemented by this organization and others serving comparable populations. Children in the summer as usual group were not restricted from attending structured programming during the summer, thus some families in this control group did pay for and attend structured programming on their own. Information as to whether these alternative programs offered meals was not captured. Additionally, this study utilized the DSQ and parent report to understand food/beverage consumption. While the DSQ is an accepted measure of children’s diet, a more rigorous dietary measure, such as 24-hour dietary recalls, may have collected more precise dietary consumption, portion sizes, meal locations, and meal timing information. Further, this measure did not disentangle different types of milk consumed or identify which foods were consumed at camp, which were brought from home, and which were consumed outside the camp settings. However, the DSQ allowed for daily food/beverage consumption over 14 days, which would not have been feasible with 24-hour dietary recalls. Strengths of this study include the randomized design, large sample size, and use of an existing community program of which families may have familiarity with and the ability to continue to use after the study is over.

## Conclusion

This RCT showed offering free SDC vouchers alone did not substantially impact the overall food/beverage intake of children from families with low income. However, on days children attended a structured summer program, particularly for longer than half days, they consumed more healthful foods/beverages, compared to days they did not attend. These findings highlight the importance of not only access, but also attendance and sustained engagement in structured environments to support healthful dietary patterns during the summer. Future research should identify additional barriers to enrollment, attendance, and healthful dietary food access and intake in summer to develop comprehensive interventions that effectively promote participation and improve summer dietary behaviors among children.

## Supplementary Information

Supplementary Material 1.

## Data Availability

Data sets generated during the current study are available from the corresponding author upon reasonable request.
