# Supplementary material for: Free summer programming on elementary-aged children’s food and beverage consumption: a randomized clinical trial
Source: Int J Behav Nutr Phys Act. 2025 Dec 20;22:160. doi: 10.1186/s12966-025-01861-1 (PMC12751208; doi:10.1186/s12966-025-01861-1)
Supplement: Supplementary file 1 — Supplementary Material 1. [file 12966_2025_1861_MOESM1_ESM.docx]

**Supplemental Figure 1.** Example Weekly Menu for Summer Day Camp.

| **Meal** | **Monday** | **Tuesday** | **Wednesday** | **Thursday** | **Friday** |
| --- | --- | --- | --- | --- | --- |
| Breakfast | Blueberry Muffin  String Cheese  Fruit Juice | Chicken Biscuit  Apple Slices | Cinni Mini’s  Fresh Fruit | Breakfast Pizza  Fresh Fruit | Pop Tart Breakfast Kit  Fresh Fruit |
| Lunch | Hamburger  Steamed Broccoli  Fresh Peach | Chicken Alfredo  Garlic Breadstick  Kale Salad  Fresh Cut Watermelon | Beefy Nachos w/ Queso  Salsa Cup  Cucumber Cup w/ Ranch  Fresh Apple | Pizza  Carrot Cup w/ Ranch  Fresh Strawberries | Deli Sub  Celery Cup w/ Ranch  Fresh Orange |
| Snack | Goldfish or Cheez-it Crackers or Chips or Popcorn or Rice Crispy Treat | | | | |

Daily milk offering at each meal: 1% White or Fat Free Chocolate
